# Angiotensin Receptor Blocker is Associated with a Lower Fracture Risk: An Updated Systematic Review and Meta-Analysis

**DOI:** 10.1155/2022/7581110

**Published:** 2022-07-14

**Authors:** Jing Wu, Mei Wang, Man Guo, Xin-Yi Du, Xiao-Zhen Tan, Fang-Yuan Teng, Yong Xu

**Affiliations:** ^1^Department of Endocrinology and Metabolism, The Affiliated Hospital of Southwest Medical University, Luzhou, Sichuan 646000, China; ^2^Cardiovascular and Metabolic Diseases Key Laboratory of Luzhou, The Affiliated Hospital of Southwest Medical University, Luzhou, Sichuan 646000, China; ^3^Sichuan Kidney Disease Clinical Medicine Research Center, The Affiliated Hospital of Southwest Medical University, Luzhou, Sichuan 646000, China; ^4^Metabolic Vascular Disease Key Laboratory of Sichuan Province, The Affiliated Hospital of Southwest Medical University, Luzhou, Sichuan 646000, China; ^5^Nephrology Department, Ziyang First People's Hospital, Ziyang, Sichuan 641300, China

## Abstract

**Background:**

Angiotensin-converting enzyme inhibitors (ACEIs) and angiotensin receptor blockers (ARBs) are widely used in the treatment of hypertension. Hypertension is often accompanied by osteoporosis. However, the relationship between ACEI/ARB and fractures remains controversial. The purpose of this meta-analysis was to update the potential relationship between ACEI/ARB and fractures.

**Methods:**

This meta-analysis was identified through PubMed, EMBASE, Cochrane Library, and Web of Science. Related studies about ACEI/ARB with the risk of fracture were published from inception to June 2022.

**Results:**

Nine qualified prospective designed studies, involving 3,649,785 subjects, were included in this analysis. Overall, the RRs of ACEI compared with the nonusers were 0.98 (95% CI: 0.88, 1.10; *P* < 0.001) for composite fractures and 0.96 (95% CI: 0.87, 1.05; *P*=0.048) for hip fractures; the RRs of ARB compared to the nonusers were 0.82 (95% CI: 0.73, 0.91; *P* < 0.001) for composite fractures and 0.85 (95% CI: 0.74, 0.97; *P*=0.028) for hip fractures. Furthermore, in the subgroup analysis, male may benefit from ARB (RR = 0.65, 95% CI: 0.49, 0.89, *P*=0.028), and the European may also benefit from ARB (RR = 0.86, 95% CI: 0.80, 0.93, *P*=0.015).

**Conclusions:**

ACEI usage will not decrease the risk of osteoporosis fracture. On the contrary, ARB usage can decrease the risk of total fracture and hip fracture, especially for males and Europeans. Compared with ACEI, for patients at higher risk of fracture in cardiovascular diseases such as hypertension, the protective effect of ARB should be considered.

## 1. Introduction

With worldwide growth in aging populations, the number of people suffering from osteoporosis is also increasing and brings a battery of physical and mental pain and enormous economic burdens to the individual and society. Fracture, especially hip fracture, is a serious complication of osteoporosis. Hip fractures often result in paralysis and loss of self-care, and there was a twenty percent increase in the mortality rate during the first year [1]. At present, known factors associated with the incidence of fractures include physical age [2], smoking [3], alcohol consumption [4], and physical exercise [5]. Besides, it is worth mentioning that the relationship between long-term drug use and fracture risk may require more attention.

The renin-angiotensin-aldosterone system (RAAS) is a pressure-boosting regulatory system produced by the kidneys in the body that can produce angiotensin II (Ang II), thereby raising blood pressure. RAAS blockers have been widely used in patients with hypertension [6] and mainly contain the angiotensin-converting enzyme inhibitor (ACEI) and angiotensin receptor blocker (ARB) in clinical practice. Strikingly, hypertension is also a common chronic disease that often presents with osteoporosis [7]. Since hypertension often coexists with osteoporosis and the widespread use of RAAS blockers for antihypertensive treatment, comprehending the effect of RAAS blockers on fracture has great clinical significance.

The relationship between RAAS blockers and bone health, structure, and metabolism has received increasing attention, but the answer remains uncertain yet [8–10]. Abuohashish 9 showed that ACEI has a protective effect in estrogen-deficient osteoporotic rats. Meanwhile, Chen [9] reported that ARB can increase bone mass via suppressing RANKL-induced ERK1/2 phosphorylation. On the contrary, Yang [10] showed that captopril, one of the angiotensin-converting enzyme inhibitors, can poison the bone of normal mice. Before us, one meta-analysis has discussed the relationship between RASS blockers and fracture risk [11]. The previous meta-analysis has shown that both ACEI and ARB use was not associated with long-term risk of composite fractures; however, both ACEI and ARB were beneficial for hip fractures. But a careful search found that three recently published studies all produced different results from the previous paper [12–14]. In view of the controversy over the role of ACEI/ARB in fracture risk, we included three new research papers in the existing meta-analysis and conducted an updated meta-analysis, and found that the results were different from the previous meta-analysis; that is, the use of ACEI was not significantly correlated with fracture risk, but ARB use was associated with a lower risk of fracture. Therefore, in clinical work, for patients with hypertension combined with higher risk of fracture, compared with ACEI, ARB should be given priority because of its lower risk of fracture.

## 2. Methods

### 2.1. Search Strategy

The literature search for articles published from inception to June 2022 was conducted in MEDLINE (via PubMed), EMBASE, Cochrane Library, and Web of Science. Mesh and free text terms used for the search were combined with methodological filters. All searches were limited to the English language, and studies were conducted with healthy humans aged 18 years.

The following search terms were used: “angiotensin-converting enzyme inhibitors,” “angiotensin II receptor blocker,” “angiotensin receptor blockers,” “inhibitors, ACE,” “ACEI,” “benazepril,” “captopril,” “enalapril,” “ARB,” “valsartan,” “irbesartan,” “losartan”; AND: “fracture,” “osteoporosis,” “fall injuries,” “bone mineral density,” “BMD,” “bone loss”(Supplement Table 1). In order to find any missing studies, we also searched the reference lists of the full-text papers and reviewed studies in all relevant publications. Moreover, we also searched Google Scholar, World Cat Dissertations, conference abstracts of the American Society for Bone and Mineral Research, and the proceedings of the International Osteoporosis Foundation World Conference on Osteoporosis. The literature search process is based on the PRISMA form. Titles and abstracts were to read and filtered by two independent reviewers, and relevant feasible articles were obtained in full. Any disagreements or uncertainties were discussed and resolved by the third investigator when needed.

### 2.2. Study Eligibility Criteria and Selection

All studies included were in accordance with PICO principles, and five eligibility criteria were as follows:

1. Adults over the age of 18 years;
2. Take any dose of ACEI/ARB intervention for at least 3 months;
3. The control group was given the placebo;
4. Provide fracture reports and risk estimates, such as relative risks (RRs), odds ratios (ORs), hazard ratios (HRs), or other measures that could be transformed into RRs, with 95% confidence intervals (CIs);
5. Only cohort studies were considered.

If different papers came from the same cohort, the paper with the most comprehensive design was brought into this analysis. Those citations both reviewers deemed irrelevant were excluded. Citations with disagreement were also included for a full review.

### 2.3. Study Quality

As shown in Supplement Table 2, the quality of the final selected studies was assessed using the Newcastle–Ottawa scale (NOS), a nine-point scale to assess the quality of cohort studies.

### 2.4. Data Extraction

Two reviewers independently used data extraction tables to extract data from various included studies. The following items were evaluated and extracted: author and year of publication; methods (study design, mean follow-up years, geographic area); participants (sample size, age range, and gender); intervention type (ACEI, ARB); fracture site; and the effect size. For subgroup analysis, we also stratified the data according to restriction factors, such as fracture site, sample size, location, gender, and quality level.

### 2.5. Statistical Analysis

RRs were used as a common measure of the association between ACEI/ARB use and fracture risk [15]. In this meta-analysis, both ORs and HRs were transformed into RRs [16, 17]. For the best explanation of the heterogeneity between these studies, we used a random-effect model to calculate RRs and 95% Cis [18]. Heterogeneity was assessed with both the *Q* statistic and the *I*^2^ index [19]. Use forest plots to show the impact of each study on the overall results. Since most studies did not specify the dose or duration of ACEI/ARB use in their original reports, subgroup analysis by these variables was not performed. Sensitivity analyses were then performed for those studies with the best available evidence. A funnel plot was used to detect publication bias, and Begg's test and Egger's tests were applied to measure funnel plot asymmetry. All the studies were conducted with Stata version 13, and statistical significance was set at a *P* value of 0.05 or less.

## 3. Results

**Figure 1:**
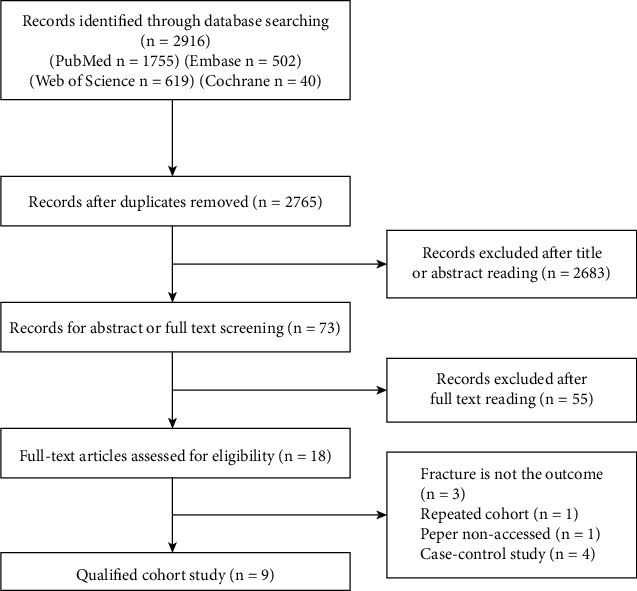
Search strategy and selection of studies.

A total of 2916 potentially relevant citations were obtained through the all-sided search. As a result, we included 9 cohort studies [12–14, 20–25]. Appraisal of the original studies is shown in Supplementary Table 1. The quality score ranged from 6 to 9. Our meta-analysis provides fracture data regarding 3,649,785 individuals. Details of the literature search and study selection flow based on the PRISMA statement are shown in Figure 1. The general characteristics of the studies included in the meta-analysis are summarized in Table 1.

### 3.1. Study Characteristics

The studies were from different regions: three from America [12, 20, 25], three from Europe [13, 22, 23], and three from Asia [14, 21, 24]. The time of follow-up durations for the qualified studies ranged from 1 to 11 years, and the sample size ranged from 1144 to 1,586,554. Fractures were identified through medical records, imaging reports, questionnaires, or administrative data. The most frequent confounders, such as age, sex, and body mass index (BMI), were adjusted in these studies.

### 3.2. ACEI/ARB and the Risk of Composite Fracture

**Figure 2:**
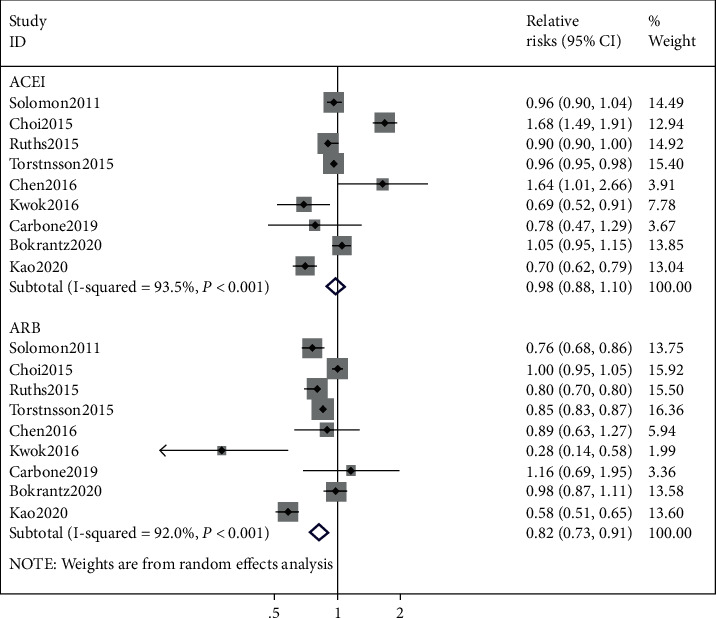
ACEI/ARB and the risk of composite fracture.

The RR (95% CI) of ACEI/ARB usage associated with composite fractures is summarized in Figure 2. Our meta-analysis showed that the usage of ACEI was not associated with a reduced risk of composite fractures (RR = 0.98, 95% CI 0.88, 1.10; *I*^2^ = 93.5%, *P* < 0.001). However, the risk of composite fractures was significantly reduced in subjects receiving ARB compared to nonusers (RR = 0.82, 95% CI 0.74–0.92, *I*^2^ = 91.2%, *P* < 0.001).

### 3.3. ACEI/ARB and Risk of Hip Fracture

**Figure 3:**
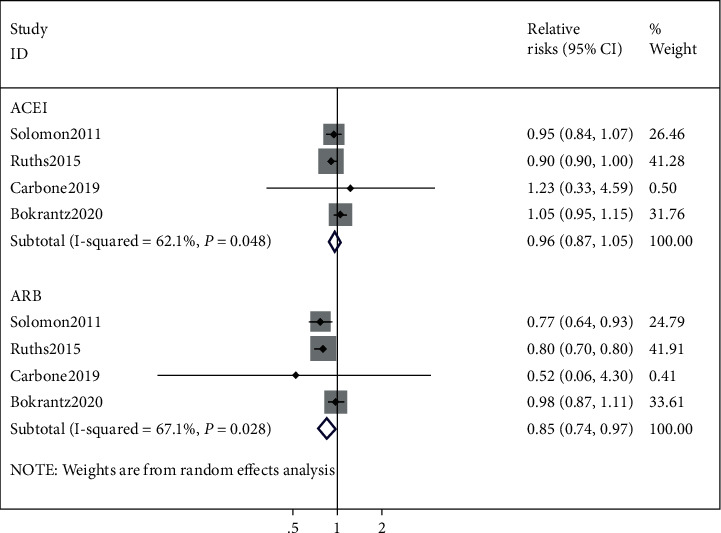
ACEI/ARB and the risk of hip fracture.

The effects of ACEI/ARB on the risk of hip fracture are summarized in Figure 3. When hip fracture was included as the only outcome measurement, however, the ACEI usage also cannot decelerate the risk of hip fracture (RR = 0.96, 95% CI 0.87–1.05, *I*^2^ = 62.1%, *P*=0.048). In contrast, the usage of ARB would reduce the risk of hip fracture as compared to nonusers (RR = 0.85, 95% CI 0.74, 0.97; *I*^2^ = 67.1%; *P*=0.028).

### 3.4. Subgroup and Sensitivity Analysis

The relative risk of fracture associated with ACEI/ARB usage by subgroups is summarized in Supplement Table 3 and Supplement Table 4. We conducted subgroup analysis in terms of the fracture site, sample size, study location, gender, and quality level. Interestingly, male may benefit from ARB blockers (RR = 0.65, 95% CI 0.49–0.89, *I*^2^ = 71.9%, *P*=0.028). What's more, when grouped by regions, the European may benefit from ARB blockers (RR = 0.86, 95% CI 0.80–0.93, *I*^2^ = 76.1%, *P*=0.015). However, in the female subgroup, the ACEI/ARB usage was not statistically significant. ACEI usage did not have a positive effect on fracture in any of the groups. In the grouping of sample size and quality level, both the combined results of five large-sample size cohort studies [12, 20–23] and five high-quality original studies (quality score ≥ 7) [12–14, 23, 25] have shown that ACEI cannot decelerate the risk of fracture, whereas ARB use with lower fracture risk. Unfortunately, most studies do not address drug doses or the effects of ACEI/ARB on other fracture sites (vertebral, wrist), which makes further analysis difficult. A sensitivity analysis about ACEI use showed that the exclusion of anyone study from the pooled analysis did not substantially vary the results (Supplement Figure 1). The sensitivity analysis about ARB use showed that two studies [21, 23] were not covered (Supplement Figure 2). When the two studies were excluded, the results did not change (RR = 0.78, 95% CI 0.74, 0.82; I^2^ = 87.5%, *P* < 0.001).

### 3.5. Publication Bias

Publication bias is initially examined by drawing funnel plots. Then, check it further with Begg's test and Egger's test. For ACEI, ARB and ACEI or ARB use, the Begg's test for publication bias was not significant (P = 0.754, P = 0.754 and P = 0.917). For ACEI, ARB and ACEI or ARB use, the Egger's test was also not significant (P = 0.823, P = 0.553 and P = 0.583). Additionally, the use of the trim and fill correction procedure did not alter the results. No evidence of publication bias was detected.

## 4. Discussion

We conducted a comprehensive meta-analysis, including data from 9 qualified prospective cohort studies investigating the association between ACEI/ARB use and the risk of fracture. We found that ACEI use was not significantly associated with fracture risk, but ARB use was associated with a lower risk of fracture. Besides, by the subgroup analysis, males and European may benefit from ARB.

Our updated meta-analysis differs in several important ways from the previous review performed by Kunutsor et al. [11]. First, our meta-analysis involved more fracture events compared to the previous meta-analysis, as it combines the most recently published evidence on the topic to date. This study has more significant statistical power and adds three newly published studies with different results from previous ones. Furthermore, data from previous meta-analyses combined with hip fractures included only two studies. In contrast, we have added two new studies [12, 13] on hip fracture to make the results more reliable. This may have some influence on drug selection in clinical practice. In addition, we found that previous reports did not perform careful subgroup analyses, and in order to explore possible sources of heterogeneity in more detail than previously reported, subgroup analysis by sex and region showed that long-term use of ARB was associated with a greater reduction in fracture risk in men and the European population.

The mechanisms underlying the beneficial effects of RAAS inhibitors on bone fracture remain debated in recent years. Apart from the more well-known systemic RAAS activations, the tissue RAAS activation also plays an important role via the endocrine effects, which mediate important physiological stimuli including bone and osteoporosis [26]. A growing number of in vivo studies have shown that tissue RAAS may result in osteoporosis. This phenomenon might via affecting the RANKL/RANK/OPG system to adjust bone metabolism [27–29]. Strikingly, tissue RAAS activity may also exert several other well-known osteoporosis risk factors or treatment modalities. Vitamin D inhibits renin gene transcription and suppresses RAAS activity [30]. Serum vitamin D level was negatively correlated with circulating RAAS activity [31, 32]. Besides, regular exercise could prevent tissue RAAS and inhibit future osteoporosis [33–35]. On the contrary, obesity may increase tissue RAAS activity [36].

Some mechanisms may explain the difference between ACEI and ARB. First, the activation of RASS can increase the plasma concentration level of Ang II, which leads to a series of physiological effects. Although ACEI and ARB share the same mechanism pathway (renin-angiotensin-aldosterone system blocking), neither ACEI nor ARB can completely inhibit the production of Ang II and the toxic effects of Ang II [37]. The relative efficacy of ACEI in suppressing Ang II levels was less than that of ARB during chronic treatment [38]. Alternative enzymatic pathways bypassing ACEI may produce angiotensin II, which will be blocked by ARB [39]. Therefore, the ability of ACEI to maintain a consistent suppression of plasma and tissue Ang II levels is limited [40]. Second, the classical actions of Ang II are mediated by binding to the angiotensin II type 1 (AT1) receptors and angiotensin II type 2 (AT2) receptors [41]. AT1 receptors play a dominant role in the known actions of Ang II [42]. ARB can only reduce AT1 receptor activity, while ACEI can block multiple receptor activities including AT1 and AT2 receptors [43]. In some ways, however, the AT2 receptor can counteract several effects initiated by the AT1, although the mechanism remains unclear [44]. Third, compared with ARB, the mechanism of action of ACEI is more complex. ACEI is known to cause dry cough and angioedema [45] as it can inhibit bradykinin degradation [46]. Studies have shown that bradykinin, as a mediator of inflammation, can decrease osteoblasts differentiation and increase osteoclasts formation [47]. Thus, ACEI may increase the risk of fracture by increasing bradykinin levels. In the future, the different effects of ACEI and ARB need to be further explored.

The subgroup analysis has shown that men would benefit from the ARB use, but it was not statistically significant for women. This may be because women's bone metabolism and bone density are more affected by estrogen than ARB [48], which has a smaller effect on fracture risk. Europeans would also benefit from the ARB use, which may be related to genes, diet, and lifestyle in different regions, but more research is needed. Our sensitivity analysis and subgroup analysis revealed that the differences in sample size and geographic region included in the original studies were major sources of heterogeneity. These results suggest that only long-term use of an ARB may be associated with a lower incidence of fracture, especially in men and Europeans.

We minimize the sources of heterogeneity by strictly implementing inclusion and exclusion criteria and excluding low-quality studies. A random-effect model was used to eliminate some heterogeneity. Subgroup analysis and sensitivity analysis were used to explore the source of heterogeneity. There was significant heterogeneity in this study, which may be due to different dosage, regimen, duration, and other reasons. In addition, bone metabolism is also affected by the gender and age of the population, and postmenopausal women and elderly people are more prone to fracture, so the ratio of gender and age will also lead to the generation of heterogeneity. Heterogeneity may be caused by whether the subjects take drugs that affect bone metabolism such as vitamin D and calcium tablets, as well as unclear conditions such as sun exposure and exercise.

This study also has several limitations. First, many of the studies suffer from significant sources of bias, and the effect in many occasions was assessed by very few studies. Second, due to the limited information we gathered from the original studies, we were unable to obtain treatment time and dose data for further analysis. Third, none of the included studies mentioned bone metabolism indexes and bone mineral density, so these aspects were not analyzed. Fourth, several drugs are often used concurrently in clinical, making it challenging to assess specific drug effects. So more high-quality studies are needed.

## 5. Conclusion

In conclusion, this meta-analysis demonstrated that ACEI cannot decelerate the risk of fracture, whereas ARB has a protective effect on fracture risk. Hence, in clinical practice, ARB may be a priority when patients suffer from hypertension with a higher risk of fracture.

## Figures and Tables

**Table 1:** Characteristics of studies on the association of ACEI/ARB with the risk of fracture.

Study	Year	Country	Baseline age (year)	Men (%)	Follow-up (years)	Sample size	Fracture site	Effect size (CI 95%)	Adjustment main
Solomn [20]	2011	USA	≥65	22.9	1	376061	All, hip, wrist, Humerus and pelvic	All: ACEI:0.96 (0.90–1.04) ARB: 0.76 (0.68–0.86) hip: ACEI: 0.95 (0.84–1.07) ARB: 0.77 (0.64–0.93)	Age, gender, race, carlson comorbidity score, number of physician visits, acute-care hospitalizations, number of different medications, osteoporosis diagnoses and medications, prior fractures, BMD testing, use of medications with fracture associations.
Choi [21]	2015	South Korea	≥50	48.6	1.9	528522	All, vertebral, And non-vertebral	ACEI: 1.68 (1.49–1.91) ARB: 1.00 (0.95–1.05)	Age, gender, comorbidity score, diabetes, osteoporosis, osteoporosis treatment, and osteoporosis-related diseases.
Ruths [22]	2015	Norway	72.8	44	5.2	906422	Hip	ACEI: 0.90 (0.90–1.00) ARB: 0.80 (0.70–0.80)	NA
Torstensson [23]	2015	Denmark	≥65	81.2	6.7	1586554	All	ACEI: 0.96 (0.95, 0.98) ARB: 0.85 (0.83, 0.87)	Age, gender, calendar year, comorbidities and exposure to the other classes of CVD-drugs.
Chen [24]	2016	Taiwan	65–80	43.6	11	1144	All	ACEI: 1.64 (1.01–2.66) ARB: 0.89 (0.63–1.27)	Age, sex, comorbidities, and concurrent medication.
Kwok [25]	2016	USA	≥65	100	6.8	2573	Non-vertebral, Hip or wrist	ACEI: 0.69 (0.52, 0.91) ARB: 0.28 (0.14, 0.58)	Age, tricyclic antidepressants, thiazide use, previous fracture, inability to complete a narrow walk trial, falls in previous year, depressed mood, hip BMD, DM, cardiac failure, hypertension, duration of use of loop diuretic, statin and beta blocker.
Carbone [12]	2019	USA	50–79	0	6.5	131793	All, hip, Upper limb, lower limb, central body	All: ACEI: 0.78(0.47, 1.29) ARB: 1.16(0.69, 1.95) hip: ACEI: 1.23(0.33, 4.59) ARB: 0.52(0.06, 4.30)	Age, BMI, physical function, total calcium and vitamin D, race/ethnicity, smoking status at baseline, parental history of hip fracture, history of fracture after age 55, reported health status, history of diabetes, CVD, hypertension, alcohol use at baseline, region, medication use at baseline.
Bokrantz [13]	2020	Swedish	≥50	43.3	7	59246	Hip	ACEI: 1.05(0.95–1.15) ARB: 0.98(0.87–1.11)	Age, sex, BMI, smoking and SBP level, concurrent medicate, drugs, ethnicity/origin, educational level, and level of income.
Kao [14]	2020	Taiwan	>45	58.6	6	57470	All	ACEI: 0.70(0.62–0.79) ARB: 0.58(0.51–0.65)	NA

## Data Availability

The data used to support the findings of this study are included within the article and available from the corresponding author upon request.

## Abbreviations

RAAS: Renin-angiotensin-aldosterone system
ACEI: Angiotensin-converting enzyme inhibitor
ARB: Angiotensin receptor blocker; Ang II: angiotensin II
RRs: Relative risks
ORs: Odds ratios
HRs: Hazard ratios
CIs: Confidence intervals
NOS: Newcastle–Ottawa scale
AT1: Angiotensin II type 1
AT2: Angiotensin II type 2.

## Supplementary Materials

Supplementary Materials Supplement Table 1. Search Strategy. Supplement Table 2. Quality score used by the Newcastle–Ottawa scale (NOS). Supplement Table 3. Subgroup analysis (ACEI). Supplement Table 4. Subgroup analysis (ARB). Supplement Figure 1. Sensitivity analysis (ACEI). Supplement Figure 2. Sensitivity analysis (ARB).
